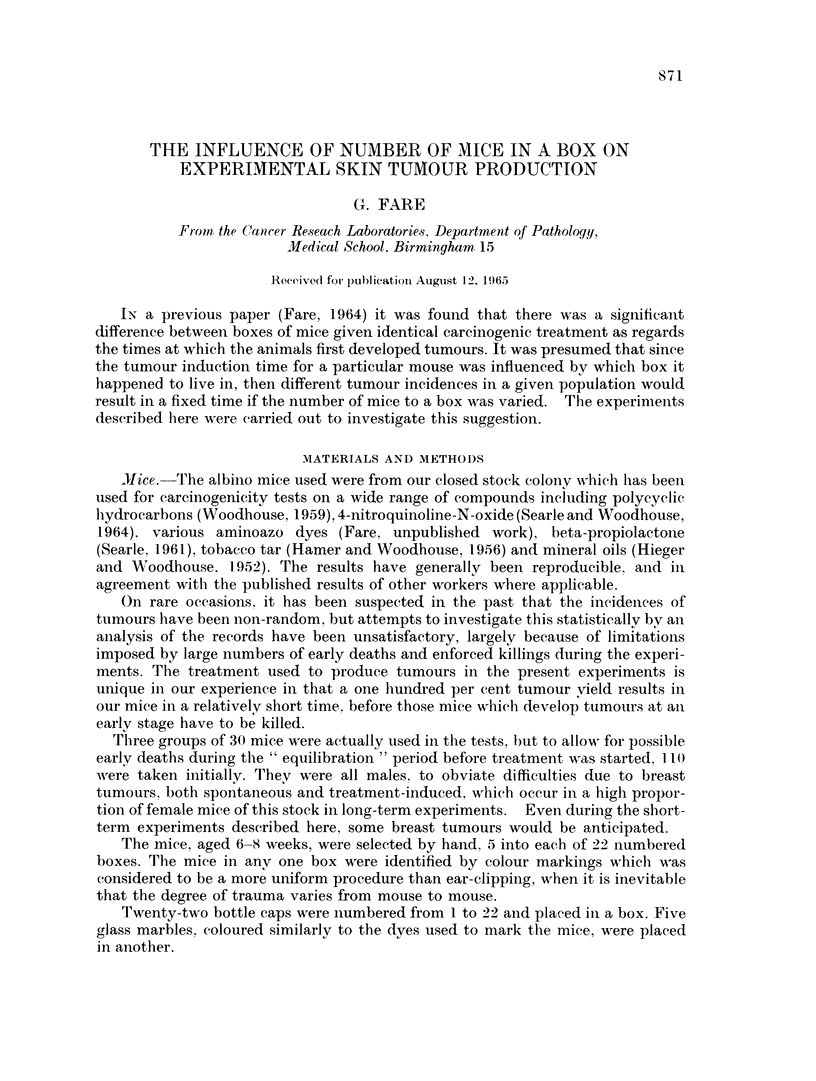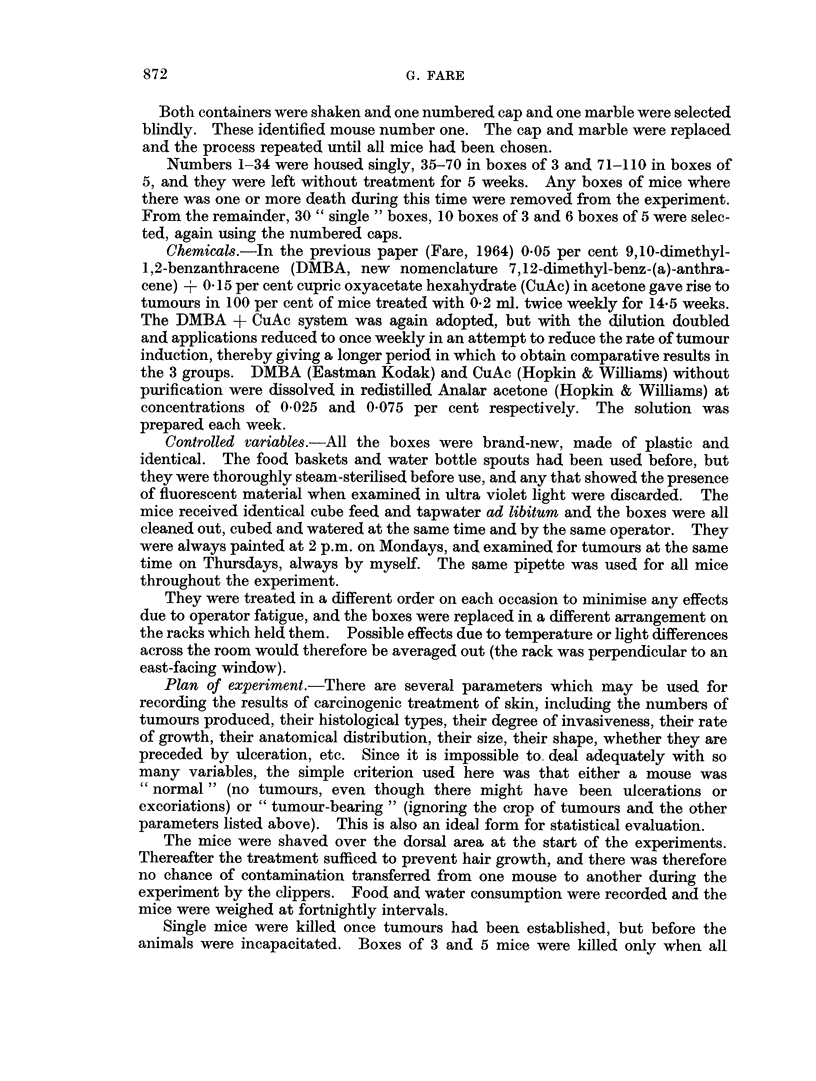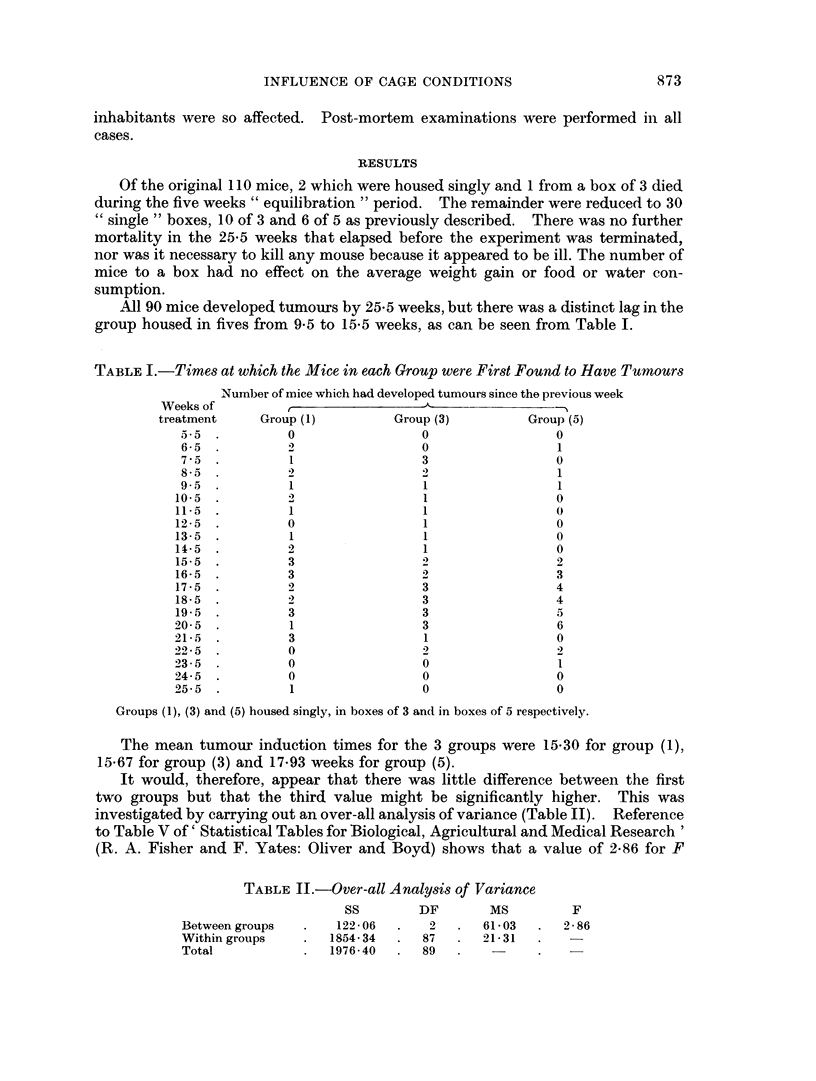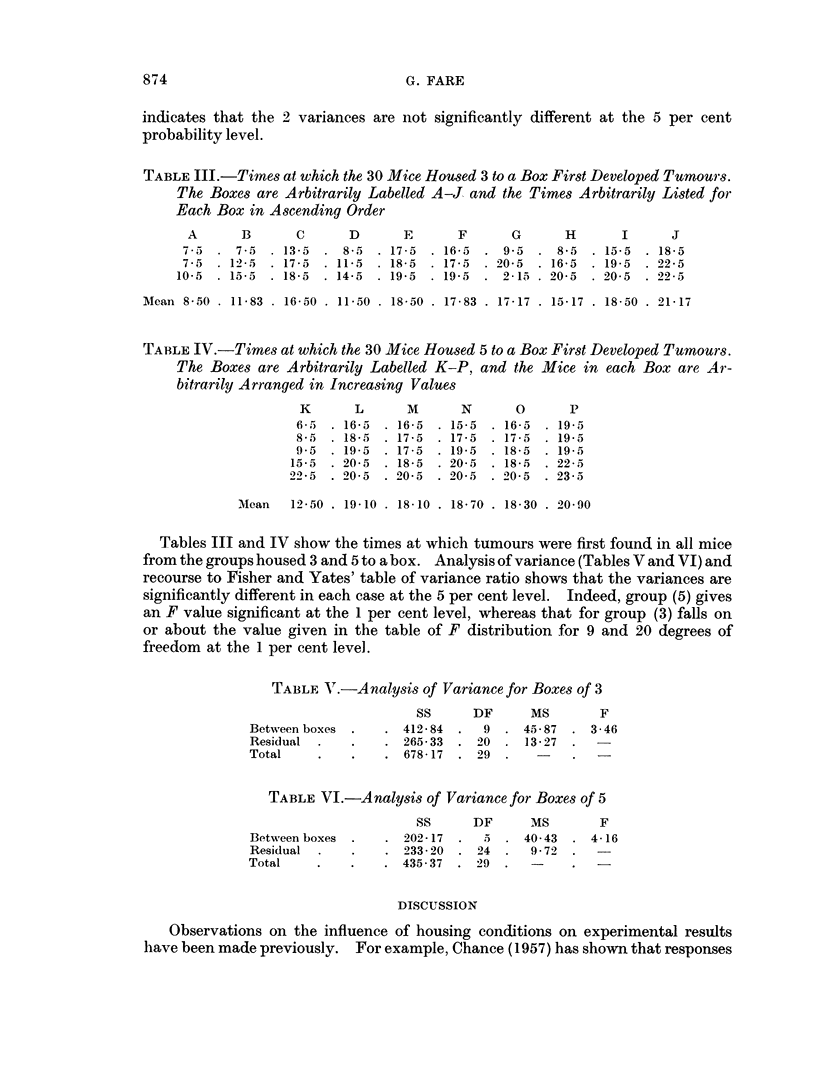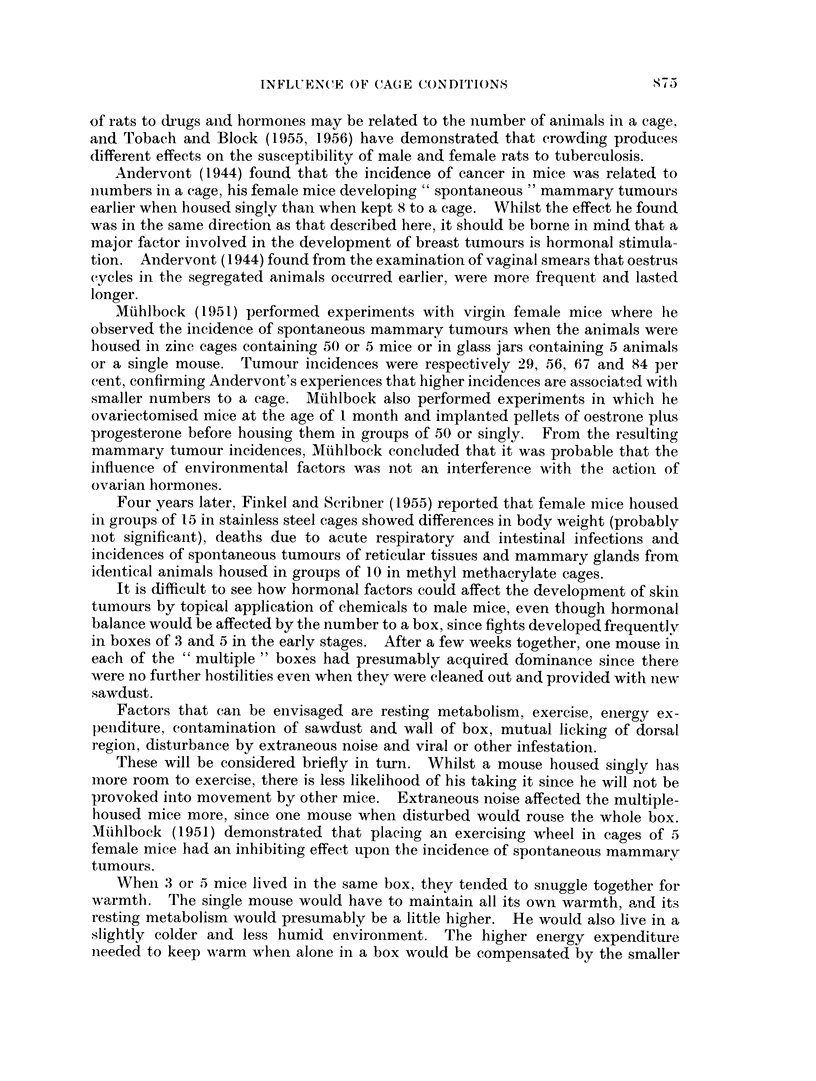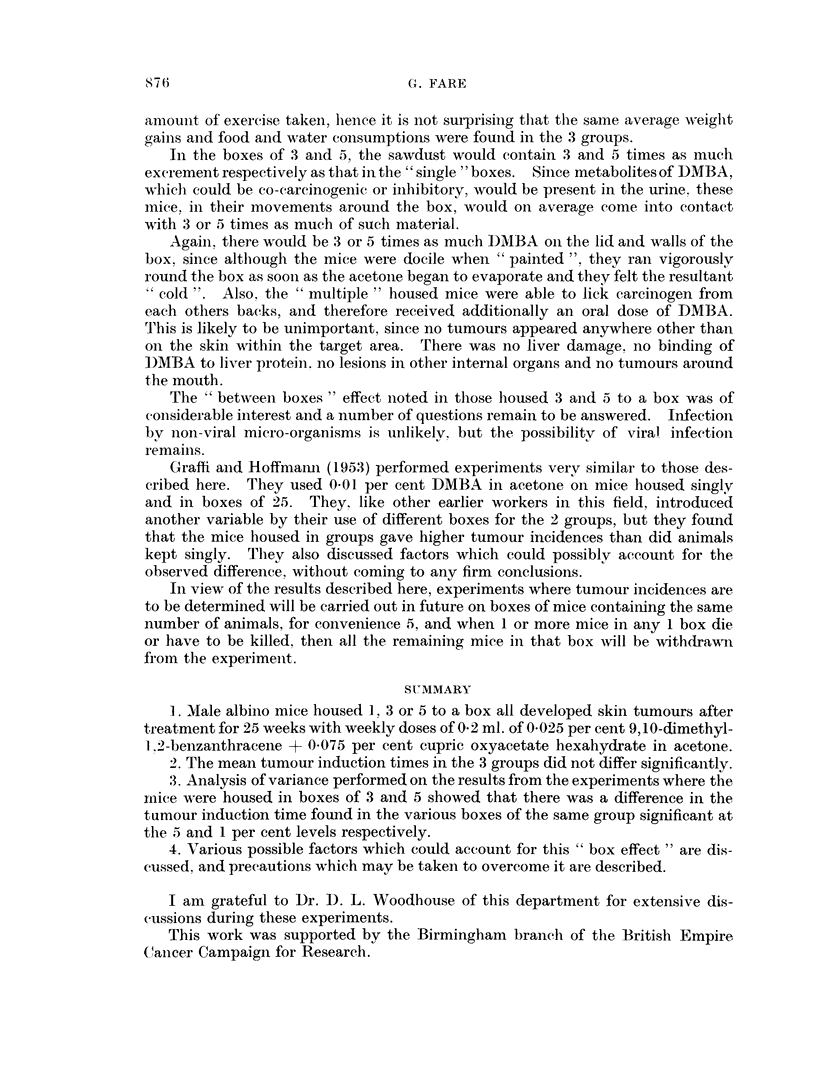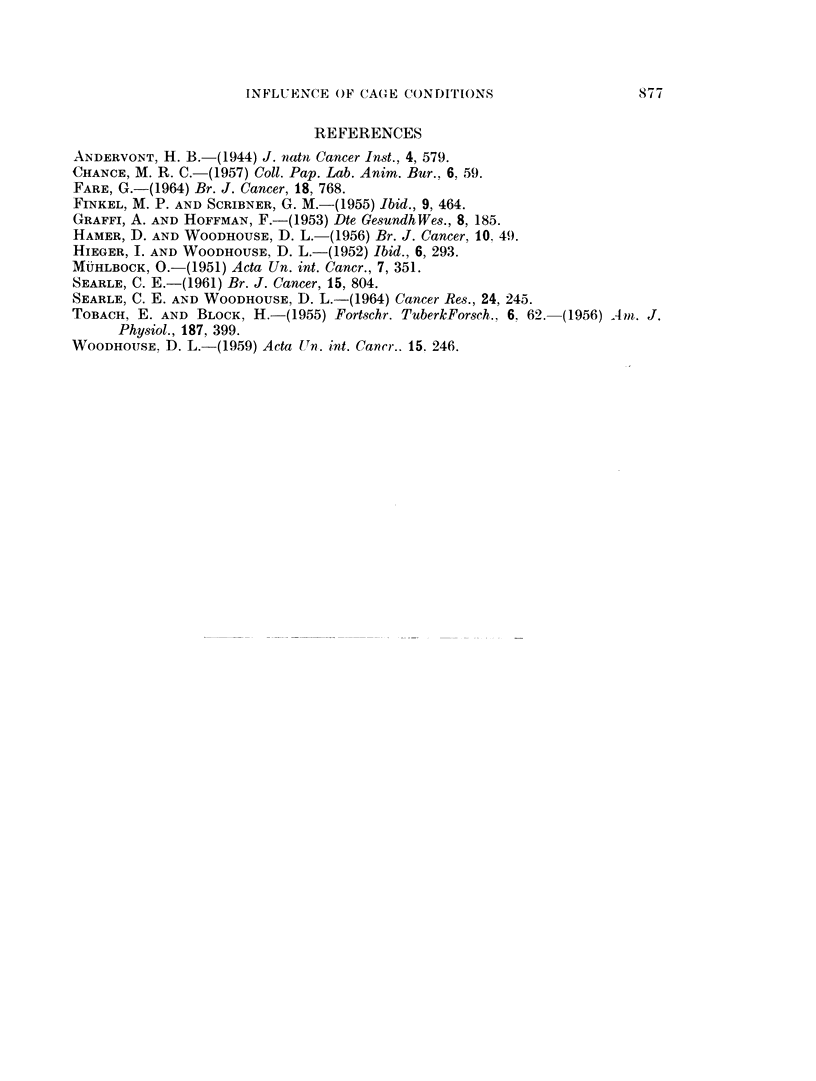# The influence of number of mice in a box on experimental skin tumour production.

**DOI:** 10.1038/bjc.1965.101

**Published:** 1965-12

**Authors:** G. Fare


### THE INFLUENCE OF NUMBER OF MICE IN A BOX ON EXPERIMENTAL SKIN TUMOUR PRODUCTION

G. FARE

*From the Cancer Reseach Laboratories, Department of Pathology, Medical School, Birmingham 15*

Received for publication August 12, 1965

In a previous paper (Fare, 1964) it was found that there was a significant difference between boxes of mice given identical carcinogenic treatment as regards the times at which the animals first developed tumours. It was presumed that since the tumour induction time for a particular mouse was influenced by which box it happened to live in, then different tumour incidences in a given population would result in a fixed time if the number of mice to a box was varied. The experiments described here were carried out to investigate this suggestion.

### MATERIALS AND METHODS

*Mice.*—The albino mice used were from our closed stock colony which has been used for carcinogenicity tests on a wide range of compounds including polycyclic hydrocarbons (Woodhouse, 1959), 4-nitroquinoline-N-oxide (Searle and Woodhouse, 1964), various aminoazo dyes (Fare, unpublished work), beta-propiolactone (Searle, 1961), tobacco tar (Hamer and Woodhouse, 1956) and mineral oils (Hieger and Woodhouse, 1952). The results have generally been reproducible, and in agreement with the published results of other workers where applicable.

On rare occasions, it has been suspected in the past that the incidences of tumours have been non-random, but attempts to investigate this statistically by an analysis of the records have been unsatisfactory, largely because of limitations imposed by large numbers of early deaths and enforced killings during the experiments. The treatment used to produce tumours in the present experiments is unique in our experience in that a one hundred per cent tumour yield results in our mice in a relatively short time, before those mice which develop tumours at an early stage have to be killed.

Three groups of 30 mice were actually used in the tests, but to allow for possible early deaths during the " equilibration " period before treatment was started, 110 were taken initially. They were all males, to obviate difficulties due to breast tumours, both spontaneous and treatment-induced, which occur in a high proportion of female mice of this stock in long-term experiments. Even during the short-term experiments described here, some breast tumours would be anticipated.

The mice, aged 6–8 weeks, were selected by hand, 5 into each of 22 numbered boxes. The mice in any one box were identified by colour markings which was considered to be a more uniform procedure than ear-clipping, when it is inevitable that the degree of trauma varies from mouse to mouse.

Twenty-two bottle caps were numbered from 1 to 22 and placed in a box. Five glass marbles, coloured similarly to the dyes used to mark the mice, were placed in another.

Both containers were shaken and one numbered cap and one marble were selected blindly. These identified mouse number one. The cap and marble were replaced and the process repeated until all mice had been chosen.

Numbers 1–34 were housed singly, 35–70 in boxes of 3 and 71–110 in boxes of 5, and they were left without treatment for 5 weeks. Any boxes of mice where there was one or more death during this time were removed from the experiment. From the remainder, 30 " single " boxes, 10 boxes of 3 and 6 boxes of 5 were selected, again using the numbered caps.

*Chemicals.*—In the previous paper (Fare, 1964) 0·05 per cent 9,10-dimethyl-1,2-benzanthracene (DMBA, new nomenclature 7,12-dimethyl-benz-(a)-anthracene) + 0·15 per cent cupric oxyacetate hexahydrate (CuAc) in acetone gave rise to tumours in 100 per cent of mice treated with 0·2 ml. twice weekly for 14·5 weeks. The DMBA + CuAc system was again adopted, but with the dilution doubled and applications reduced to once weekly in an attempt to reduce the rate of tumour induction, thereby giving a longer period in which to obtain comparative results in the 3 groups. DMBA (Eastman Kodak) and CuAc (Hopkin & Williams) without purification were dissolved in redistilled Analar acetone (Hopkin & Williams) at concentrations of 0·025 and 0·075 per cent respectively. The solution was prepared each week.

*Controlled variables.*—All the boxes were brand-new, made of plastic and identical. The food baskets and water bottle spouts had been used before, but they were thoroughly steam-sterilised before use, and any that showed the presence of fluorescent material when examined in ultra violet light were discarded. The mice received identical cube feed and tapwater *ad libitum* and the boxes were all cleaned out, cubed and watered at the same time and by the same operator. They were always painted at 2 p.m. on Mondays, and examined for tumours at the same time on Thursdays, always by myself. The same pipette was used for all mice throughout the experiment.

They were treated in a different order on each occasion to minimise any effects due to operator fatigue, and the boxes were replaced in a different arrangement on the racks which held them. Possible effects due to temperature or light differences across the room would therefore be averaged out (the rack was perpendicular to an east-facing window).

*Plan of experiment.*—There are several parameters which may be used for recording the results of carcinogenic treatment of skin, including the numbers of tumours produced, their histological types, their degree of invasiveness, their rate of growth, their anatomical distribution, their size, their shape, whether they are preceded by ulceration, etc. Since it is impossible to deal adequately with so many variables, the simple criterion used here was that either a mouse was " normal " (no tumours, even though there might have been ulcerations or excoriations) or " tumour-bearing " (ignoring the crop of tumours and the other parameters listed above). This is also an ideal form for statistical evaluation.

The mice were shaved over the dorsal area at the start of the experiments. Thereafter the treatment sufficed to prevent hair growth, and there was therefore no chance of contamination transferred from one mouse to another during the experiment by the clippers. Food and water consumption were recorded and the mice were weighed at fortnightly intervals.

Single mice were killed once tumours had been established, but before the animals were incapacitated. Boxes of 3 and 5 mice were killed only when all inhabitants were so affected. Post-mortem examinations were performed in all cases.

### RESULTS

Of the original 110 mice, 2 which were housed singly and 1 from a box of 3 died during the five weeks " equilibration " period. The remainder were reduced to 30 " single " boxes, 10 of 3 and 6 of 5 as previously described. There was no further mortality in the 25·5 weeks that elapsed before the experiment was terminated, nor was it necessary to kill any mouse because it appeared to be ill. The number of mice to a box had no effect on the average weight gain or food or water consumption.

All 90 mice developed tumours by 25·5 weeks, but there was a distinct lag in the group housed in fives from 9·5 to 15·5 weeks, as can be seen from Table I.

TABLE I.—*Times at which the Mice in each Group were First Found to Have Tumours*

| Weeks of treatment | Number of mice which had developed tumours since the previous week | | |
|---|---|---|---|
| | Group (1) | Group (3) | Group (5) |
| 5·5 | 0 | 0 | 0 |
| 6·5 | 2 | 0 | 1 |
| 7·5 | 1 | 3 | 0 |
| 8·5 | 2 | 2 | 1 |
| 9·5 | 1 | 1 | 1 |
| 10·5 | 2 | 1 | 0 |
| 11·5 | 1 | 1 | 0 |
| 12·5 | 0 | 1 | 0 |
| 13·5 | 1 | 1 | 0 |
| 14·5 | 2 | 1 | 0 |
| 15·5 | 3 | 2 | 2 |
| 16·5 | 3 | 2 | 3 |
| 17·5 | 2 | 3 | 4 |
| 18·5 | 2 | 3 | 4 |
| 19·5 | 3 | 3 | 5 |
| 20·5 | 1 | 3 | 6 |
| 21·5 | 3 | 1 | 0 |
| 22·5 | 0 | 2 | 2 |
| 23·5 | 0 | 0 | 1 |
| 24·5 | 0 | 0 | 0 |
| 25·5 | 1 | 0 | 0 |

Groups (1), (3) and (5) housed singly, in boxes of 3 and in boxes of 5 respectively.

The mean tumour induction times for the 3 groups were 15·30 for group (1), 15·67 for group (3) and 17·93 weeks for group (5).

It would, therefore, appear that there was little difference between the first two groups but that the third value might be significantly higher. This was investigated by carrying out an over-all analysis of variance (Table II). Reference to Table V of ' Statistical Tables for Biological, Agricultural and Medical Research ' (R. A. Fisher and F. Yates: Oliver and Boyd) shows that a value of 2·86 for *F*

TABLE II.—*Over-all Analysis of Variance*

| | SS | DF | MS | F |
|---|---|---|---|---|
| Between groups | 122·06 | 2 | 61·03 | 2·86 |
| Within groups | 1854·34 | 87 | 21·31 | — |
| Total | 1976·40 | 89 | — | — |

indicates that the 2 variances are not significantly different at the 5 per cent probability level.

TABLE III.—*Times at which the* 30 *Mice Housed* 3 *to a Box First Developed Tumours. The Boxes are Arbitrarily Labelled A–J and the Times Arbitrarily Listed for Each Box in Ascending Order*

| | A | B | C | D | E | F | G | H | I | J |
|---|---|---|---|---|---|---|---|---|---|---|
| | 7·5 | 7·5 | 13·5 | 8·5 | 17·5 | 16·5 | 9·5 | 8·5 | 15·5 | 18·5 |
| | 7·5 | 12·5 | 17·5 | 11·5 | 18·5 | 17·5 | 20·5 | 16·5 | 19·5 | 22·5 |
| | 10·5 | 15·5 | 18·5 | 14·5 | 19·5 | 19·5 | 2·15 | 20·5 | 20·5 | 22·5 |
| Mean | 8·50 | 11·83 | 16·50 | 11·50 | 18·50 | 17·83 | 17·17 | 15·17 | 18·50 | 21·17 |

TABLE IV.—*Times at which the* 30 *Mice Housed* 5 *to a Box First Developed Tumours. The Boxes are Arbitrarily Labelled K–P, and the Mice in each Box are Arbitrarily Arranged in Increasing Values*

| | K | L | M | N | O | P |
|---|---|---|---|---|---|---|
| | 6·5 | 16·5 | 16·5 | 15·5 | 16·5 | 19·5 |
| | 8·5 | 18·5 | 17·5 | 17·5 | 17·5 | 19·5 |
| | 9·5 | 19·5 | 17·5 | 19·5 | 18·5 | 19·5 |
| | 15·5 | 20·5 | 18·5 | 20·5 | 18·5 | 22·5 |
| | 22·5 | 20·5 | 20·5 | 20·5 | 20·5 | 23·5 |
| Mean | 12·50 | 19·10 | 18·10 | 18·70 | 18·30 | 20·90 |

Tables III and IV show the times at which tumours were first found in all mice from the groups housed 3 and 5 to a box. Analysis of variance (Tables V and VI) and recourse to Fisher and Yates' table of variance ratio shows that the variances are significantly different in each case at the 5 per cent level. Indeed, group (5) gives an $F$ value significant at the 1 per cent level, whereas that for group (3) falls on or about the value given in the table of $F$ distribution for 9 and 20 degrees of freedom at the 1 per cent level.

TABLE V.—*Analysis of Variance for Boxes of* 3

| | SS | DF | MS | F |
|---|---|---|---|---|
| Between boxes | 412·84 | 9 | 45·87 | 3·46 |
| Residual | 265·33 | 20 | 13·27 | — |
| Total | 678·17 | 29 | — | — |

TABLE VI.—*Analysis of Variance for Boxes of* 5

| | SS | DF | MS | F |
|---|---|---|---|---|
| Between boxes | 202·17 | 5 | 40·43 | 4·16 |
| Residual | 233·20 | 24 | 9·72 | — |
| Total | 435·37 | 29 | — | — |

### DISCUSSION

Observations on the influence of housing conditions on experimental results have been made previously. For example, Chance (1957) has shown that responses of rats to drugs and hormones may be related to the number of animals in a cage, and Tobach and Block (1955, 1956) have demonstrated that crowding produces different effects on the susceptibility of male and female rats to tuberculosis.

Andervont (1944) found that the incidence of cancer in mice was related to numbers in a cage, his female mice developing " spontaneous " mammary tumours earlier when housed singly than when kept 8 to a cage. Whilst the effect he found was in the same direction as that described here, it should be borne in mind that a major factor involved in the development of breast tumours is hormonal stimulation. Andervont (1944) found from the examination of vaginal smears that oestrus cycles in the segregated animals occurred earlier, were more frequent and lasted longer.

Mühlbock (1951) performed experiments with virgin female mice where he observed the incidence of spontaneous mammary tumours when the animals were housed in zinc cages containing 50 or 5 mice or in glass jars containing 5 animals or a single mouse. Tumour incidences were respectively 29, 56, 67 and 84 per cent, confirming Andervont's experiences that higher incidences are associated with smaller numbers to a cage. Mühlbock also performed experiments in which he ovariectomised mice at the age of 1 month and implanted pellets of oestrone plus progesterone before housing them in groups of 50 or singly. From the resulting mammary tumour incidences, Mühlbock concluded that it was probable that the influence of environmental factors was not an interference with the action of ovarian hormones.

Four years later, Finkel and Scribner (1955) reported that female mice housed in groups of 15 in stainless steel cages showed differences in body weight (probably not significant), deaths due to acute respiratory and intestinal infections and incidences of spontaneous tumours of reticular tissues and mammary glands from identical animals housed in groups of 10 in methyl methacrylate cages.

It is difficult to see how hormonal factors could affect the development of skin tumours by topical application of chemicals to male mice, even though hormonal balance would be affected by the number to a box, since fights developed frequently in boxes of 3 and 5 in the early stages. After a few weeks together, one mouse in each of the " multiple " boxes had presumably acquired dominance since there were no further hostilities even when they were cleaned out and provided with new sawdust.

Factors that can be envisaged are resting metabolism, exercise, energy expenditure, contamination of sawdust and wall of box, mutual licking of dorsal region, disturbance by extraneous noise and viral or other infestation.

These will be considered briefly in turn. Whilst a mouse housed singly has more room to exercise, there is less likelihood of his taking it since he will not be provoked into movement by other mice. Extraneous noise affected the multiple-housed mice more, since one mouse when disturbed would rouse the whole box. Mühlbock (1951) demonstrated that placing an exercising wheel in cages of 5 female mice had an inhibiting effect upon the incidence of spontaneous mammary tumours.

When 3 or 5 mice lived in the same box, they tended to snuggle together for warmth. The single mouse would have to maintain all its own warmth, and its resting metabolism would presumably be a little higher. He would also live in a slightly colder and less humid environment. The higher energy expenditure needed to keep warm when alone in a box would be compensated by the smaller amount of exercise taken, hence it is not surprising that the same average weight gains and food and water consumptions were found in the 3 groups.

In the boxes of 3 and 5, the sawdust would contain 3 and 5 times as much excrement respectively as that in the "single" boxes. Since metabolites of DMBA, which could be co-carcinogenic or inhibitory, would be present in the urine, these mice, in their movements around the box, would on average come into contact with 3 or 5 times as much of such material.

Again, there would be 3 or 5 times as much DMBA on the lid and walls of the box, since although the mice were docile when "painted", they ran vigorously round the box as soon as the acetone began to evaporate and they felt the resultant "cold". Also, the "multiple" housed mice were able to lick carcinogen from each others backs, and therefore received additionally an oral dose of DMBA. This is likely to be unimportant, since no tumours appeared anywhere other than on the skin within the target area. There was no liver damage, no binding of DMBA to liver protein, no lesions in other internal organs and no tumours around the mouth.

The "between boxes" effect noted in those housed 3 and 5 to a box was of considerable interest and a number of questions remain to be answered. Infection by non-viral micro-organisms is unlikely, but the possibility of viral infection remains.

Graffi and Hoffmann (1953) performed experiments very similar to those described here. They used 0·01 per cent DMBA in acetone on mice housed singly and in boxes of 25. They, like other earlier workers in this field, introduced another variable by their use of different boxes for the 2 groups, but they found that the mice housed in groups gave higher tumour incidences than did animals kept singly. They also discussed factors which could possibly account for the observed difference, without coming to any firm conclusions.

In view of the results described here, experiments where tumour incidences are to be determined will be carried out in future on boxes of mice containing the same number of animals, for convenience 5, and when 1 or more mice in any 1 box die or have to be killed, then all the remaining mice in that box will be withdrawn from the experiment.

### SUMMARY

1. Male albino mice housed 1, 3 or 5 to a box all developed skin tumours after treatment for 25 weeks with weekly doses of 0·2 ml. of 0·025 per cent 9,10-dimethyl-1,2-benzanthracene + 0·075 per cent cupric oxyacetate hexahydrate in acetone.

2. The mean tumour induction times in the 3 groups did not differ significantly.

3. Analysis of variance performed on the results from the experiments where the mice were housed in boxes of 3 and 5 showed that there was a difference in the tumour induction time found in the various boxes of the same group significant at the 5 and 1 per cent levels respectively.

4. Various possible factors which could account for this "box effect" are discussed, and precautions which may be taken to overcome it are described.

I am grateful to Dr. D. L. Woodhouse of this department for extensive discussions during these experiments.

This work was supported by the Birmingham branch of the British Empire Cancer Campaign for Research.

### REFERENCES

ANDERVONT, H. B.—(1944) *J. natn Cancer Inst.*, **4**, 579.
CHANCE, M. R. C.—(1957) *Coll. Pap. Lab. Anim. Bur.*, **6**, 59.
FARE, G.—(1964) *Br. J. Cancer*, **18**, 768.
FINKEL, M. P. AND SCRIBNER, G. M.—(1955) *Ibid.*, **9**, 464.
GRAFFI, A. AND HOFFMAN, F.—(1953) *Dte GesundhWes.*, **8**, 185.
HAMER, D. AND WOODHOUSE, D. L.—(1956) *Br. J. Cancer*, **10**, 49.
HIEGER, I. AND WOODHOUSE, D. L.—(1952) *Ibid.*, **6**, 293.
MÜHLBOCK, O.—(1951) *Acta Un. int. Cancr.*, **7**, 351.
SEARLE, C. E.—(1961) *Br. J. Cancer*, **15**, 804.
SEARLE, C. E. AND WOODHOUSE, D. L.—(1964) *Cancer Res.*, **24**, 245.
TOBACH, E. AND BLOCK, H.—(1955) *Fortschr. TuberkForsch.*, **6**, 62.—(1956) *Am. J. Physiol.*, **187**, 399.
WOODHOUSE, D. L.—(1959) *Acta Un. int. Cancr.*, **15**, 246.